# The Expression, Prognostic Value, and Immunological Correlation of MCEMP1 and its Potential Role in Gastric Cancer

**DOI:** 10.1155/2022/8167496

**Published:** 2022-03-26

**Authors:** Pu Huang, Yawen Liu, Baoqing Jia

**Affiliations:** ^1^Medical School of Chinese PLA, Beijing, China; ^2^Department of General Surgery, The First Medical Centre, Chinese PLA General Hospital, Beijing, China; ^3^Department of Gastroenterology, Affiliated Hospital of Jiangsu University, Jiangsu University, Beijing, China

## Abstract

**Purpose:**

Gastric cancer (GC) is a lethal cancer with a poor 5-year relative survival, which requires a new research perspective. Our study aims to explore the biological impact of the mast cell-expressed membrane protein 1 (MCEMP1) in GC, which includes its expression and potential biological functions.

**Methods:**

The expression of MCEMP1 was assessed through public databases. The GO, KEGG, and GESA analyses were conducted to explore the biofunction of MCEMP1. And ssGSEA was used to analyze the infiltration of the immune cells for MCEMP1. The proliferation, migration, and invasion of GC cells were analyzed through CCK8, colony-forming, wound healing, Transwell, and Western blot assay.

**Results:**

The expression of MCEMP1 was higher in GC tissues. Further, we found a close relationship between MCEMP1 and poorer prognosis of gastric cancer by prognostic analysis. The functional analysis showed that MCEMP1 is involved in immune, inflammation, and metabolism-related pathways. The ssGSEA analysis indicated MCEMP1 mRNA expression was associated with immune infiltration of multiple immune cells. In cellular experiments, the invasion and metastasis of gastric cancer cells could be promoted by regulating the rise of MCEMP1 expression. Western blot analysis showed that regulation of MCEMP1 expression can affect EMT-related protein expression and that NF-*κ*B expression is involved in this process.

**Conclusion:**

MCEMP1 shows a potential value for the prognosis in GC. And, abnormal expression of MCEMP1 in GC is correlated with tumor immune cell infiltration. In in vitro experiments, MCEMP1 can affect the proliferation, migration, and invasion of GC cells by regulating EMT, in which TLR4/NOD2/NF-*κ*B was involved.

## 1. Introduction

Gastric cancer, a tumor of the digestive tract, is highly recurrent worldwide, with a total of approximately 1.09 million new cases in 2020 through the Global Cancer Observatory (GCO), a cancer database [1]. Due to the lack of early and specific signs and symptoms of gastric cancer, the majority of patients are already in a progressive or even advanced stage when diagnosed and often progress rapidly. Although there are several treatment options for gastric cancer, early detection and early resection are still the best treatment for gastric cancer [2, 3]. Currently, it is believed that postoperative metastasis and recurrence are high-risk factors for the recurrence of gastric cancer. Factors such as the patient's physical condition, the treatment plan received, tumor stage, and the pathological characteristics of the tumor itself can all influence the prognosis of gastric cancer. The disadvantage of these predictive markers is that they can usually be obtained after surgery. Therefore, there is an urgent need for a preoperatively accessible marker to predict the prognostic risk of gastric cancer patients.

MCEMP1 is a protein that spans the entire length of the cell membrane, typically expressed by immune-related cells such as mast cells, macrophages, etc. [4]. The exact function of MCEMP1 has not yet been determined, but related explorations have been carried out. A study revealed that downregulation of MCEMP1 caused a decline in interleukins and interferons that inhibited monocyte proliferation and promoted apoptosis in septic mice, thereby promoting apoptosis suppressing inflammation in sepsis [5]. Several studies have also confirmed the possible involvement of MCEMP1 in regulating mast cell differentiation, immune responses, and inflammatory responses [6]. As a biomarker, it has been suggested that MCEMP1 expression in peripheral blood may contribute to the diagnosis of stroke and serve as a biomarker of a 1-month stroke prognosis. [7]. In another study on gastric cancer, MCEMP1 was also involved in constructing a predictive model as a factor on regulatory T cells [8]. Sammarco's study demonstrated that mast cells actively participate in tumor angiogenesis and lymphangiogenesis and are closely associated with the immune microenvironment of gastric cancer [9]. Based on the fact that MCEMP1 is expressed by mast cells as a transmembrane protein and the multifunctional role of mast cells in cancer, this has led to an interest in the role of MCEMP1 in gastric cancer progression.

Based on the above information, we speculate that MCMEP1 may play an essential role in gastric cancer, especially in immune-related aspects. In this study, we aim to investigate the expression, clinical relevance, function, and immune relevance of MCMEP1 in GC by a combination of bioinformatics analysis and experimental exploration.

## 2. Methods

### 2.1. Data Source (Oncomine, TCGA, GTEx, and GEO)

The RNA sequencing (RNA-seq) and clinical data (age, gender, survival time, survival status, tumor grade, TNM stage, and pathological stage) for gastric cancer and other 32 tumors were available through “UCSC XENA” (https://xenabrowser.net/datapages/) data portal [10]. The downloaded data consists of tumor tissue and normal tissue from the TCGA and GTEx databases. In addition, we also investigated the expression of MCMEP1 through the Oncomine public database and the dataset in GEO (GSE54129).

### 2.2. Enrichment Analysis

The differential expression of mRNAs was detected by the limma package. We set the filtering conditions for thresholds of differentially expressed genes (DEGs) to adjusted *P* < 0.05 and |log2 (FoldChange)| >1. To better investigate the biocharacteristics of MCEMP1 in gastric cancer, functional enrichment analysis of Gene Ontology (GO) and the Kyoto Encyclopedia of Genes and Genomes (KEGG) was performed using the “cluster profile” R package. Gene Set Enrichment Analysis (GSEA) was employed to analyze the enrichment of the dataset between the high and low expressing group, with a false discovery rate (FDR) <25% and a nominal *P* value <5% set as cut-off criteria.

### 2.3. Immune Infiltration Analysis

The single-sample Gene Set Enrichment Analysis (ssGSEA) algorithm for enrichment scoring in immune infiltration uses 24 types of immune cell markers as gene sets to calculate an enrichment score for each type of immune cell in each sample, inferring the infiltration status of immune cells in each sample [11]. To explore the correlation between prognostic gene expression immune infiltrates of GC, the cohorts were sorted into high-risk and low-risk segments based on the median MCEMP1 expression. The correlation between individual molecules and the immune cell fraction of each sample was analyzed using Spearman correlation analysis. Last, Statue single-sample GSEA (ssGSEA) was implemented via the “gsva” R package [12].

### 2.4. Cell Lines and Cell Culture

Four human gastric cancer cell lines BGC-823, HGC-27, SGC-7901, and MGC-803, and human gastric normal epithelial cell line, GES-1, were included in this experiment. BGC-823, HGC-27, and GES-1 cells were cultured with RPMI-1640 medium (HyClone, Beijing, China), and SGC-7901 and MGC-803 cells were used with DMEM medium (HyClone, Beijing, China). 10% fetal bovine serum (Gibco, Carlsbad, CA, USA), 100 units/ml penicillin, and 100 mg/ml streptomycin (Solarbio, Beijing, China) were added to the cell culture medium as essential components. The cell growth environment was set at 37°C in a 5% CO2 humidified incubator.

### 2.5. Plasmid Construction

The RT-PCR with primers MCEMP1-all-F: 5′-GGAATTCGCCACCATGCAAGCAC CAGCCTTCAGGG-3′; and MCEMP1-all-R: 5′-GCTCTAGATTATTGAGGTGAGG ACTGTG-3′, to extend the entire MCEMP1 sequence. The EcoRI and XhaI positions of the FV026-Entry plasmid (Fubio) were then inserted and conjugated to the vector. The MCEMP1 shRNA 1-3 oligos (MCEMP1 shRNA1-F, 5′-GATCGGTCTCAG CCAAGAATCAAGGCTCGAGCCTTGATTCTTGGCTGAGACCTTTTTT-3′; MCEMP1 shRNA1-R, 5′-AATTAAAAAAGGTCTCAGCCAAGAATCAAGGCTCG AGCCTTGATTCTTGGCTGAGACC-3′; MCEMP1 shRNA2-F, 5′-GATCGCATCAT CCTGTCAGCCTTCACTCGAGTGAAGGCTGACAGGATGATGCTTTTTT-3′; MCEMP1 shRNA1-R, 5′-AATTAAAAAAGCATCATCCTGTCAGCCTTCACTCGA GTGAAGGCTGACAGGATGATGC-3′; MCEMP1 shRNA3-F, 5′-GATCGGGTGA ACGGCTGTGTCATTACTCGAGTAATGACACAGCCGTTCACCCTTTTTT-3′; MCEMP1 shRNA3-R, 5′-AATTAAAAAAGGGTGAACGGCTGTGTCATTACTCG AGTAATGACACAGCCGTTCACCC-3′) was initially annealed into a double strand and was then replicated onto the FV055-puro vector (Fubio).

### 2.6. Cell Transfection and Generation of Stably Transfected Cell Lines

The transfection reagent involved in the transfection of MCEMP1-OE or shRNA plasmids into cells was Lipofectamine 2000 (Invitrogen, Carlsbad, CA, USA). Stably transfected cell lines were procured by transfection of MCEMP1 shRNA, packaging plasmid, and envelope plasmid into HEK293T cells. The virus was harvested after 48 hours and infected into the cells.

### 2.7. Cell Counting Kit-8 (CCK-8) Proliferation Assay

96-well plates were selected for seeding cells with 1000 cells per well per group and cultured in a suitable external environment. On days 1, 2, 3, and 4, the original medium in each well was replaced by 100 *μ*l of medium and 10 *μ*l of CCK-8 solution. One hour after incubation in the adapted incubator, the cells will be measured for absorbance at 450 mm (OD450).

### 2.8. Real-Time PCR Assay

For the experiments to isolate total RNA, we chose RNAiso Plus (Takara). For the cDNA in the reverse transcription experiments, we picked the RevertAid First Strand cDNA Synthesis Kit (Thermo). Then, the real-time PCR based on SYBR green in triplicate is carried out by using the CFX-96 sequence detection system (Bio-Rad). The primers were as follows: *β*-actin, 5′-TGGCACCCAGCACAATGAA-3′ (forward), 5′-CTAAGTCATAGTCCGCCTAGAAGCA-3′ (reverse); MCEMP1, 5′-CAGGGACAAGAAACAGGGGG-3′ (forward), 5′-GGTCGTGAATGACCACCCTT-3′. (reverse).

### 2.9. Western Blotting Assay

With cold PBS flushing of the cultured cells, and then load in 1 x SDS buffer processing under 100°C for 10 minutes, samples were centrifuged at 12,000 rcf for 5 minutes; then, approximately 10 *μ*l of protein was added to each electrophoresis channel lane and finally separated and shifted onto PVDF membranes. The obtained samples were sealed with skimmed milk mixture 5% for one hour at room temperature and then hatched with the primary antibody at 4°C for 8–12 h. Membranes that have been incubated with the primary antibody are rinsed with configured TBS-T buffer (10 mM Tris-HCl, pH 7.4, 150 mM NaCl, 0.05% Tween 20) and finally incubated with horseradish peroxidase-conjugated secondary antibody. The antibodies were mouse anti-*β*-Tubulin (Cell Signaling, No 6181), rabbit anti-N-Cadherin (No 13116), and rabbit anti-E-Cadherin (No 3195) which were from Cell Signaling. The antibodies were rabbit anti-MCEMP1 (ab188572), rabbit anti-TLR4 (ab13556), human anti-NOD2 (ab31488), and rabbit anti-NF*κ*B (ab32360) which were from Abcam.

### 2.10. Colony Formation Assay

Transfected cells were raised on six-well plates for 14 days. The cells were removed from the incubation tank, and the cells were immobilized in 4% paraformaldehyde for 15 minutes, followed by crystal violet staining for 30 minutes. After the staining is completed, we use the camera to record the appearance of the colony and calculate the number of colonies per hole.

### 2.11. Wound Healing Assay

After treatment, the cells were inoculated in 24-well plates and incubated until fully fused. Next, the culture fluid is aspirated, and a small gap is cut in the cell layer with a thin tube to create a model of a wounded cell layer. The extent of wound healing was measured by recording the wound closure distance with a digital camera after the cells had been grown in a serum-free medium for one whole day.

### 2.12. Transwell Assays

The Transwell insert, with a permeable pore space of 8 *μ*m (Corning, Corning, NY, USA), was selected for the following experiments. The treated cells are placed into the upper chamber filled with a serum-free medium instead of using the serum-containing medium in the lower layer. After a full day of cell culture, the cells were harvested and fixed in 4% paraformaldehyde for 1/4 hour and finally colored with crystalline violet. The crystalline violet was washed off before image acquisition, and the insert was dried. The protocol for the cell invasion assay is mainly identical to that of the cell migration assay, with the difference being the overlay of BD Matrigel in the Transwell (BD Bioscience, Corning, NY, USA).

### 2.13. Statistical Methods

For statistical comparisons between the two sets of nominal variables, we used the chi-square test or Fisher's exact probability test. The Wilcoxon test was used for comparison between the two groups for ordered variables, while the *t*-test was considered for statistical analysis for continuous variables. In order to assess and compare patient survival, the Kaplan-Meier method opted for us. The Log-rank test or Cox proportional hazards model was used to compare the survival curves of the two groups according to whether the critical prognostic factors were balanced (comparisons were made using logit tests or Cox proportional hazards models). The spss20.0 software generated the results of the analysis. A two-sided test tested all the statistics. Statistically, significant comparisons between groups were defined as when the *P* value was less than or equal to 0.05.

## 3. Results

In order to systematically analyze the importance of MCMEP1 in gastric cancer, we first analyzed the relative expression of MCMEP1 in tumor versus normal tissues validated by different tumor databases, as well as cell lines. Furthermore, through clinical correlation analysis of public database tumor data and bioinformatics-related functional analysis, we found that MCMEP1 is highly associated with gastric cancer, especially in terms of immunity. Next, we identified an essential role for MCEMP1 in promoting proliferation, colony formation, migration, and invasion of gastric cancer cells. Finally, through Western blotting experiments, we found that MCEMP1 induced EMT in GC cells, possibly through the TLR4/NOD2/NF-*κ*B pathway.

### 3.1. The mRNA Expression Level of Mast Cell Expressed Membrane Protein 1 (MCEMP1) in Human Cancers

**Figure 1:**
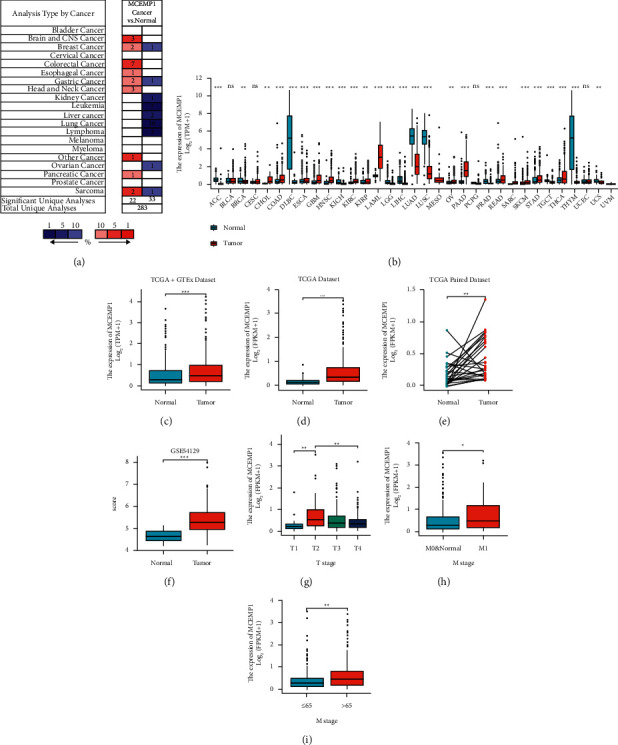
MCEMP1 expression in human cancers. (a) The mRNA level of MCEMP1 in various types of cancer (Oncomine); (b) MCEMP1 expression in different cancer types based on the GTEx and TCGA database; (c) the expression of MCEMP1 was different between GC and normal gastric tissues from GTEx databases (Normal 210 patients, Tumor 414 patients) and (d) TCGA (Normal 32 patients, Tumor 375 patients); (e) expression of MCEMP1 in TCGA-paired gastric cancer and normal tissues (Normal 27 patients, Tumor 27 patients); (f) the aberrant expression of MCEMP1 is based on the GEO database (GSE54129, Normal 21 patients, Tumor 111 patients); (g) MCEMP1 expression in different T-staging patients (T1 : T2 : T3 : T4 = 19 : 80 : 168 : 100) and (h) in different M-staging patients (M0&Normal: M1 = 330 : 32) and (i) in different age patients (207 patients >65 y, 164 patients ≤65 y). ^*∗*^*P* < 0.05, ^*∗∗*^*P* < 0.01, ^*∗∗∗*^*P* < 0.001.

To compare the mRNA expression of MCEMP1 in human tumors, we went through the Oncomine database. The results demonstrated that MCEMP11 was highly expressed in gastrointestinal tumors, including colon cancer, esophageal cancer, and gastric cancer (Figure 1(a)). Because the TCGA database has limited adjacent normal tissues of gastric cancer, we fused the data from TCGA and GTEx databases. Then, we synthesized the expression levels of the MCEMP1 gene in the dataset. The analysis revealed that MCEMP1 expression was significantly upregulated in various tissues versus normal tissues (Figure 1(b)). In detail, TCGA and GTEx data combined, TCGA separate datasets, and TCGA-paired datasets all have significant differences (Figures 1(c)–1(e)).

Similarly, in the GEO cohort GSE54129, we obtained the same conclusion that MCMEP1 was relatively more expressed in tumor tissues (Figure 1(f)). Besides, we discovered that MCEMP1 was also correlated with the age and pathological stages (T stage, M stage) of patients with GC (Figures 1(g)–1(i)).

### 3.2. Prognostic Value of MCEMP1 in Gastric Cancer

**Figure 2:**
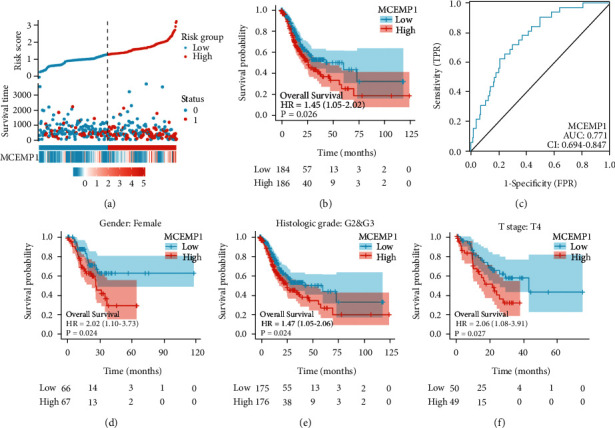
Prognostic value of MCEMP1 in gastric cancer based on TCGA database. (a) MCEMP1 expression distribution, survival status, and the MCEMP1 expression profiles heatmap. (b) Survival curve of MCEMP1 expression. (c) ROC curve of MCEMP1 expression. (d) Survival curve of MCEMP1 expression in a subgroup of females and (e) in a subset of histologic grade: G2 and G3 and (f) in a subset of T4 stage.

To evaluate the association between MCEMP1 expression and prognosis of gastric cancer, we assessed the interaction between MCEMP1 expression levels and patient survival using TCGA-related data. In particular, a negative trend of MCEMP1 expression in relation to overall survival (OS) was observed in gastric cancer patients. In addition, our comprehensive evaluation by risk score plot, survival status, and Kaplan-Meier survival analysis concluded that the higher the MCEMP1 expression, the worse the survival of the patients (Figures 2(a) and 2(b)). The area under the curve (AUC) evaluated by the prediction model (receiver operating characteristic curve, ROC) exceeded 0.75, proving that MCEMP1 has an excellent predictive value (Figure 2(c)). Besides, we further assessed the survival outcome in different T-staging, sex, and the histologic grade with GC patients. As shown in Figures 2(d)–2(f), the high expression level of MCEMP1 in the subgroup of females, T4 stage, G2, and G3 histologic grade is also related to poor overall survival.

### 3.3. Functional Enrichment Analysis

**Figure 3:**
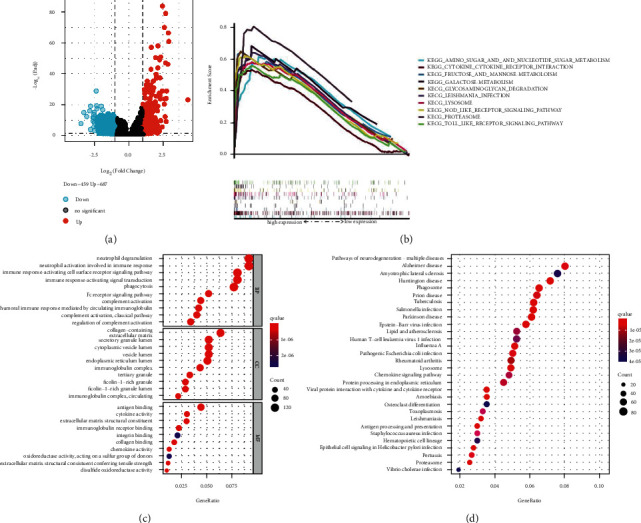
Differentially expressed genes (DEGs) and functional enrichment analysis. (a) Volcano plot of DEGs between samples with high MCEMP1 expression and low MCEMP1 expression. (b) GSEA for samples with high MCEMP1 expression. (c) GO enrichment analysis. (d) KEGG enrichment by MCEMP1 expression-correlated upregulated DEGs.

To further analyze the possible biological processes involved in MCEMP1 in gastric cancer, we proposed enriching the data of TCGA by GO, KEGG. After analysis by “limma” R package operation, 1146 DEGs were sought out between tumor tissues and adjacent nontumorous tissues (*P* value <0.05, logFC >1). The volcano map shows that 687 of the DEGs are upregulated, and 459 are downregulated (Figure 3(a)). The GSEA, KEGG, and GO analyses were adopted to discuss the potential biological mechanism related to DEGs. The GSEA analysis shows that twenty-two gene sets of the upregulated DEGs group are significant at FDR <25%, and 19 gene sets are significantly enriched at nominal *P* value <5%. More specifically, the enrichment analysis reveals that the upregulated DEGs group was enriched in the NOD-like receptor signaling pathway, TOLL-like receptor signaling pathway, and metabolism-related biological pathways, like amino sugar, nucleotide sugar, galactose, fructose, mannose, etc. (Figure 3(b)). In addition, The GO enrichment analysis showed a strong correlation between the upregulated DEGs and immune response, such as neutrophil activation involved in immune response, immune response-activating cell surface receptor signaling pathway, immunoglobulin complex, circulating, and immunoglobulin receptor binding (Figure 3(c)). Moreover, the upregulated DEGs were subject to KEGG pathway enrichment analysis. In Figure 3(d), we can see that the enriched pathways include neurodegeneration multiple diseases, Alzheimer's disease, amyotrophic lateral sclerosis, Huntington disease, salmonella infection, tuberculosis, lipid, and atherosclerosis T-cell leukemia virus 1 infection.

### 3.4. Relationship between MCEMP1 Expression and Immune Cell Infiltration in GC

**Figure 4:**
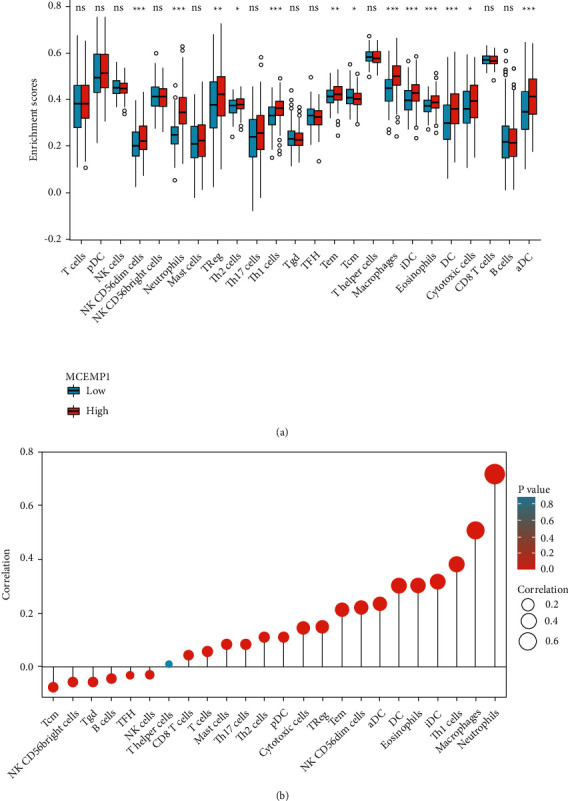
Relationship between MCEMP1 expression and immune cell infiltration in GC. (a) Enrichment scores for 24 immune cell types between high and low MCEMP1 expression group. (b) Relationships among infiltration levels of 24 immune cell types and MCEMP1 expression profiles by Spearman's analysis.

MCEMP1, as a mast cell, is closely associated with the immune infiltration of tumor cells. To investigate the link between MCMEP1 and immunity, we proposed correlating the immune-related data in TCGA by the ssGSEA method. First, we quantified and analyzed the correlation between MCEMP1 and the level of immune cell (24 species) infiltration by using the ssGSEA method. In Figure 4(a), we can observe a remarkable direct connection between MCEMP1 expression and the infiltration levels of neutrophils, macrophages, and Th1 cells. In addition, we measured the enrichment scores of 24 immune-related cells to discuss the correlation between immune cell types and risk scores. In comparing immune cell types and risk scores, we found that NK CD56dim cells, neutrophils, T helper 1 (Th1 cells), macrophages, eosinophils, immature Dendritic Cells (iDC), and activated DC (aDC) were significantly high in the high-MCEMP1 group, suggesting that the high-MCMEP1 group may be more involved in regulating the immune and inflammatory response (Figure 4(b)).

### 3.5. MCEMP1 Expression Validation in Cell Lines and Plasmid Construction

**Figure 5:**
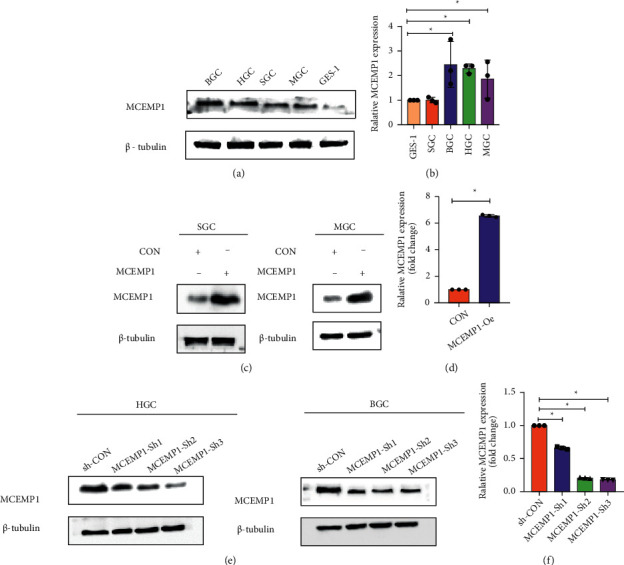
MCEMP1 expression validation and plasmid construction in gastric cancer cell lines. (a) The relative expression levels of MCEMP1 in different pancreatic cell lines (BGC, HGC, SGC, MGC, and GES-1) were measured by Western blot and (b) real-time PCR. (c) Expression of the plasmid oe-MCEMP1 was analyzed in SGC and MGC cells, and oe-con was used to track the expression of oe-MCEMP1, validation by Western blot and (d) real-time PCR. (e) Protein and (f) mRNA levels of MCEMP1 in HGC and BGC cells transfected with sh-MCEMP1 plasmids and sh-con plasmid. ^*∗*^*P* < 0.05.

The expression profile of MCMEP1 has been verified through public databases in the previous section. To further analyze the expression of MCEMP1 in tumors, as well as to prepare for the next step of cellular functional assays, we propose determining the target genes in cell lines and constructing target gene-related knockdown and overexpression cell lines. First, we examine the MCMEP1 expression in 4 GC cell lines (BGC, HGC, SGC, and MGC) using the PCR method. The analysis revealed that MCEMP1 expression was higher in BCG, HGC, and MGC compared with normal gastric epithelial cell line GES-1 (Figure 5(b)). Similarly, Western blot experiments verified the same conclusion (Figure 5(a)). In addition, we constructed plasmid MCEMP1 overexpression systems in SGC and MGC cell lines. The PCR and Western blotting indicated that the plasmid construction was also verified to be successful, and the target gene expression was significantly higher in the MCEMP1-oe group than in the control group (Figures 5(c) and 5(d)). Relatively, sh-MCEMP1 and sh-CON were transferred into HGC and BGC cells. The analysis of protein and mRNA showed that the expression level of MCEMP1 was significantly reduced in the sh-MCEMP1 group compared to the control group, with MCEMP1-sh2 and MCEMP1-sh3 interfering more efficiently (Figures 5(e) and 5(f)).

### 3.6. MCEMP1 Promotes the Proliferation and Colony-Forming Abilities of Gastric Cancer Cells

**Figure 6:**
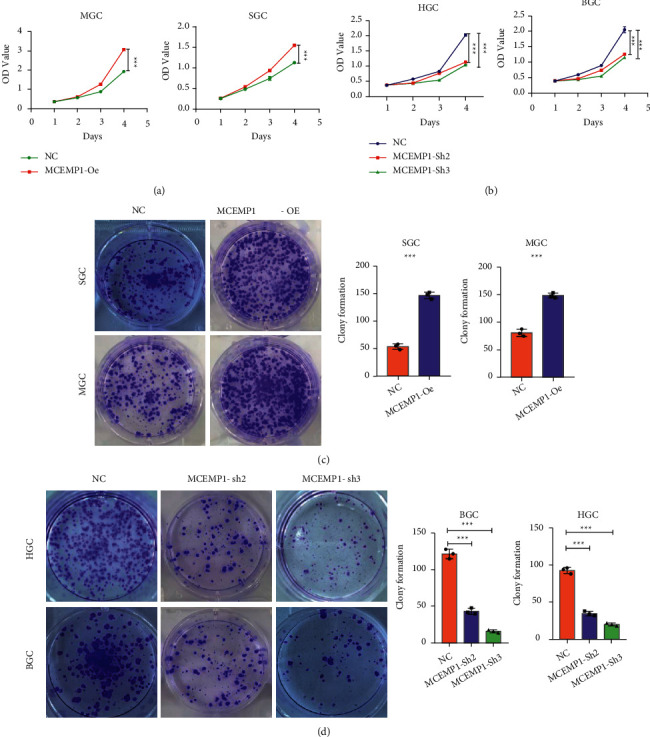
MCEMP1 promotes the proliferation and colony-forming abilities of gastric cancer cells. (a) Overexpressed MCEMP1 promotes the proliferation of MGC and SGC cells. (b) MCEMP1-shRNA inhibited the proliferation of HGC and BGC cells. (c) MCEMP1-OE promotes the formation of colonies in SGC and MGC cells. (d) MCEMP1-sh inhibited the formation of colonies in HGC and BGC cells. ^*∗∗∗*^*P* < 0.001.

To investigate the effect of MCEMP1 on GC cell growth, we first generated growth curves using the CCK-8 method. Our experimental data demonstrated MCEMP1 overexpression significantly promoted the proliferation of MGC and SGC cells, especially on day 4 (Figure 6(a)). Correspondingly, CCK-8 results confirmed that knockdown of MCEMP1 can inhibit the proliferation of HGC and BGC cells (Figure 6(b)). To further confirm the effect of MCEMP1 on GC cell proliferation, we assessed the colony-forming ability of these cells. In MGC and SGC cells, MCEMP1 overexpression resulted in larger colonies and higher colony density than the corresponding controls (Figure 6(c)). These results were also confirmed in HGC and BGC cells after the knockdown of MCEMP1. The knockdown group reduced the size and thickness of colonies (Figure 6(d)). The findings revealed that MCEMP1 facilitates the proliferation and colony formation of GC cells.

### 3.7. MCEMP1 Enhances the Invasion and Migration Ability of Gastric Cancer Cells

**Figure 7:**
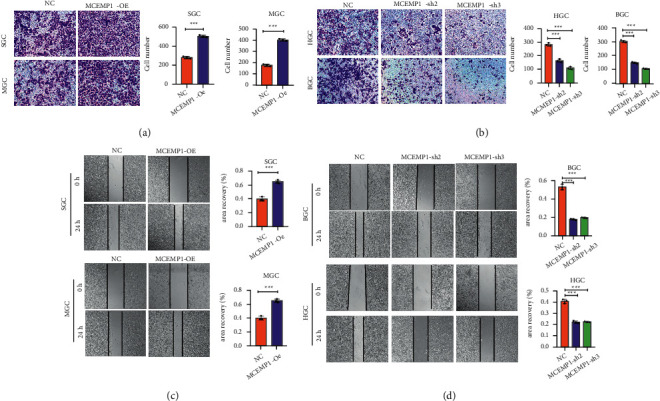
MCEMP1 enhances the invasion and migration ability of gastric cancer cells. (a) Transwell assays were used to examine the migration ability of SGC and MGC cells transfected with MCEMP1-OE and MCEMP1-CON. (b) Transwell assays were used to examine the migration ability of BGC and HGC cells transfected with MCEMP1-sh and sh-CON. (c) Wound healing assay was used to migration ability of SGC and MGC cells transfected with MCEMP1-OE and MCEMP1-CON. (d) Wound healing assay was used to determine the migration ability of BGC and HGC cells transfected with MCEMP1-sh and sh-CON. The histogram represents the statistical analysis of the wound healing assays. ^*∗∗∗*^*P* < 0.001.

To assess the effect of MCEMP1 on gastric cancer cells, including the ability of tumor cells to infiltrate and metastasize in vitro experiments, Transwell and wound healing assays were used. As shown in Figure 7(a), the wound healing assay demonstrated that overexpression of MCEMP1 accelerated the healing process. The Transwell experiment revealed that the amount of migrating cells was markedly diminished in the low MCEMP1 group (Figure 7(b)). In addition, wound healing assays displayed that overexpressing MCEMP1 of SGC and MGC exhibited increased motility (Figure 7(c)). In comparison, MCEMP1 knockdown inhibited the migratory capacity of BGC and HGC cells (Figure 7(d)). The validation of the above experiments allows us to associate the conclusion that MCEMP1 can enhance the invasiveness and metastasis of GC cells.

### 3.8. MCEMP1 Promotes the EMT (Epithelial-Mesenchymal Transition) Process in GC Cells, in Which the NF-*κ*B Pathway Was Involved

**Figure 8:**
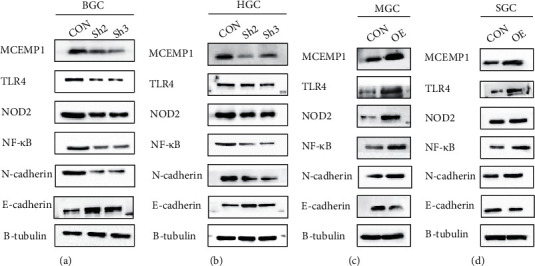
MCEMP1 promotes the EMT process in GC cells, in which the NF-*κ*B pathway was involved. (a) Western blotting was used to detect the expression of MCEMP1. TLR4, NOD2, NF-*κ*B, E-cadherin, N-cadherin, and *β*-Tubulin transfected with MCEMP1-sh and MCEMP1-control in BGC and (b) HGC cells. (c) Western blotting was used to detect the expression of MCEMP1. TLR4, NOD2, NF-*κ*B, E-cadherin, N-cadherin, and *β*-Tubulin transfected with MCEMP1-oe and MCEMP1-control in MGC and (d) SGC cells.

To investigate the influence of MCEMP1 on migration and aggression of gastric cancer cells, we assayed EMT-related markers (EMT is associated with tumor invasion and metastasis) at the protein expression level. The findings at the protein level demonstrated that MCEMP1 knockdown in gastric cancer cells resulted in the downregulation of N-cadherin and upregulation of E-cadherin, implying involvement in the EMT process. Our previous GSEA analysis showed differential gene enrichment was associated with the NOD-like receptor and TOLL-like receptor pathway. As NF-*κ*B signaling, a common downstream factor of both, is closely associated with EMT in tumors, we explored the potential effect of MCEMP1 on NF-*κ*B. We found that MCEMP1 knockdown downregulated TLR4, NOD2, and NF-*κ*b in HGC and BGC cell lines (Figures 8(a) and 8(b)). In comparison, MCEMP1 overexpression upregulated TLR4, NOD2, and NF-*κ*b (Figures 8(c) and 8(d)). The above results suggest that MCEMP1 promotes the EMT process in GC cells, in which the NF-*κ*B pathway was involved.

## 4. Discussion

To improve the prognosis of patients with gastric cancer, we need early detection, diagnosis, and individualized comprehensive treatment. However, there is still a lack of prognostic biomarkers in gastric cancer. Current research on MCEMP1 has focused on inflammation and cardiovascular-related diseases, but fewer studies have been done on tumors. Therefore, a well-rounded investigation was performed, including the expression of MCMEP1 in tumors, its prognosis, and its impact on cancer progression. EMT refers to the fact that epithelial cells are affected by some factors and then lose the tight junctions and adhesion junctions between cells [13]. At the same time, the infiltration and migration ability is enhanced, and finally, they become cells with the shape and characteristics of mesenchymal cells. Accumulating evidence has implicated EMT in various aspects of malignancy, including tumor invasion, metastases formation, and treatment resistance [14, 15]. EMT is associated with the metastatic process in numerous tumors, as well as in gastric cancer, during which epithelial cells differentiate and acquire mesenchymal features such as free cells' migration and invading distant areas [14]. Typically, we observe the EMT process characterized by the sight of an elevated N-cadherin and a reduction in E-cadherin levels [16]. In our study, overexpression of MCEMP1 increased the expression of N-cadherin but decreased the expression of E-cadherin, also suggesting that MCEMP1 may mediate gastric cancer metastasis through EMT. In functional bioinformatics studies, we identified that MCEMP1-related DEGs were mainly concentrated in metabolism, immunity, and inflammation-related pathways, including toll-like receptor and NOD-like receptor pathways. As the common downstream factor of these two pathways, Nf-*κ*b has a close relationship with them [10, 17, 18]. NF-*κ*B (nuclear factor kappa B) is a protein complex found in nuclear extracts of B lymphocytes, and its continued activation leads to uncontrolled cell growth. The NF-*κ*B signaling pathway is involved in the progression of multiple cellular responses and thus linked to a diverse range of critical bodily functions, including cell proliferation, viral infections, inflammation, and immune defense [19]. NF-*κ*B has a principal function of inhibiting apoptosis and is closely related to many processes such as tumorigenesis, growth, and metastasis [20]. Since the downstream genes of NF-*κ*B include CyclinD1 and c-Myc, the persistent activation of NF-*κ*B stimulates cell growth, leading to uncontrolled cell proliferation [21, 22]. NF-*κ*B has a significant promoting effect on tumor metastasis, and it can promote the expression of tumor metastasis-related genes VCAM-1, MMP-9, etc. [23, 24]. The two critical stages of tumor progression are cancer cell invasion and metastasis, in which NF-*κ*B-dependent genes are also involved in regulation. In addition, the growth of tumor cells and tissue infiltration requires the continuous formation of new blood vessels. The genes related to the proteins mediating new blood vessels are regulated by NF-*κ*B, including the most critical member of the angiogenic factor family—blood vessels. Endothelial cell growth factor (VEGF) [25].

Persistent activation of NF-*κ*B can enhance the transcription of the VEGF gene, thereby promoting tumor angiogenesis. A close association between EMT and NF-*κ*B activation was demonstrated in a broad range of human cancers, ranging from prostate, colon, breast, and others [26–28]. A study on lung cancer also showed that inhibition of NF-KB could inhibit the EMT process of lung cancer cells [29]. In GC cell lines, after regulation of MCEMP1, we observed the occurrence of EMT features, namely, a decrease in E-cadherin and an increase in N-cadherin protein. In addition, the expressions of TLR4, NOD2, and NF-*κ*B are also increased after overexpression of MCEMP1. We also got the same results after knocking down MCEMP1. Taken together, MCEMP1 promotes GC cell migration by promoting the EMT process involved in the NF-*κ*B signaling pathway, but its deeper mechanism remains to be studied.

The tumor microenvironment is located in a place between normal tissue and tumor tissue, and its composition is complex, including extracellular matrix, soluble molecules, and tumor stromal cells. As tumors develop, the tumor microenvironment accumulates many cells that suppress the body's immunity, such as MDSCs, regulatory T cells, tumor-associated macrophages, and a large number of inflammation-associated factors, which ultimately work together to promote tumor immune escape from tumor growth and metastasis [30–32]. Through identifying the relationship between MCMEP1 and immune cell infiltration, we identified a significantly enhanced level of infiltration of neutrophils, macrophages, Th1 cells, and DCs was clearly enhanced in the MCEMP1 high expression group. Furthermore, the expression of MCMEP1 in gastric cancer correlated notably with the immune scores of various immune cells (especially inflammation-related cells), suggesting that MCMEP1 may be involved in relevant immune processes through its involvement in inflammation-related responses, consequently, in related immune processes.

## 5. Conclusion

In summary, MCEMP1 shows a potential value for the prognosis in GC. In addition, Abnormal expression of MCEMP1 in GC is correlated with tumor immune cell infiltration. In in vitro experiments, modification of MCEMP1 can affect the invasiveness and metastasis of gastric cancer cells by regulating EMT, in which TLR4/NOD2/NF-*κ*B was involved.

## Data Availability

The datasets used and/or analyzed during the present study are available from the corresponding author upon reasonable request.
